# Visuo-Haptic Exploration for Multimodal Memory

**DOI:** 10.3389/fnint.2019.00015

**Published:** 2019-05-15

**Authors:** Alessandra Sciutti, Federica Damonte, Marta Alloisio, Giulio Sandini

**Affiliations:** ^1^Cognitive Architecture for Collaborative Technologies Unit, Istituto Italiano di Tecnologia, Genoa, Italy; ^2^DIBRIS, Università di Genova, Genoa, Italy

**Keywords:** haptic, vision, active exploration, bimodal perception, perception and action

## Abstract

When faced with a novel object, we explore it to understand its shape. This way we combine information coming from different senses, as touch, proprioception and vision, together with the motor information embedded in our motor execution plan. The exploration process provides a structure and constrains this rich flow of inputs, supporting the formation of a unified percept and the memorization of the object features. However, how the exploration strategies are planned is still an open question. In particular, is the exploration strategy used to memorize an object different from the exploration strategy adopted in a recall task? To address this question we used iCube, a sensorized cube which measures its orientation in space and the location of the contacts on its faces. Participants were required to explore the cube faces where little pins were positioned in varying number. Participants had to explore the cube twice and individuate potential differences between the two presentations, which could be performed either haptically alone, or with also vision available. The haptic and visuo-haptic (VH) exploratory strategies changed significantly when finalized to memorize the structure of the object with respect to when the same object was explored to recall and compare it with its memorized instance. These findings indicate that exploratory strategies are adapted not only to the property of the object to be analyzed but also to the prospective use of the resulting representation, be it memorization or recall. The results are discussed in light of the possibility of a systematic modeling of natural VH exploration strategies.

## Introduction

Humans are very good at recognizing objects and inferring their properties by integrating information coming from multiple sensory channels and also from motor commands. This is particularly true for haptic perception, which depends on cutaneous and kinesthetic (proprioceptive) inputs, related to the position and the force applied by the limbs used to touch, but also on the exploration strategies adopted, e.g., the velocity of the exploratory movements or the way the object is handled (Lederman and Klatzky, 2009). Recognizing an object through haptic exploration is, therefore, a multimodal ability, which requires also short term memory and a strategy to collect information (Fernandes and Albuquerque, 2012). As a result, it enables us to gather an approximate estimate of a wide range of object properties, as the weight, the size, the volume of the object at hand, together with the force needed to manipulate it. Notwithstanding this apparent complexity, it comes to us as very naturally and we use it commonly, for instance when we have to recognize the keys or a coin in a pocket or a purse.

Haptic exploration entails the acquisition of stimulus properties both in parallel, through the integration of inputs derived from the different effectors, as the fingers, the palm, the two hands; and in sequential manner, integrating information of different parts of the explored objects acquired over an extended period of time. Hence, the strategy adopted when touching or actively manipulating the object is crucial in determining the representation of the object as a whole. Starting from the seminal work by Lederman and Klatzky (1987), many authors have shown that haptic movements are specific and directed to feature evaluation, both for 3D and 2D objects (Hatwell et al., 2003; Kappers and Douw, 2011). Lederman and Klatzky (1987) have provided a systematic classification of the hand movements adopted during haptic exploration. Blindfolded participants could touch and freely explore objects of daily use to assess certain specific features. Through movement analysis and video annotation, the authors defined six stereotyped movements, each specific to determine a particular information about the object: lateral motion (texture), pressure (hardness), contour following (shape), unsupported holding (weight), enclosure (global shape), static contact (temperature). Hence, beyond individual differences, it is possible to find general models of exploratory movements tailored to extract a specific feature. For instance, to judge object texture, the majority of people will tend to touch and move on the surface, although with individual variations—e.g., using a single finger or the whole hand. Human haptic exploration is therefore specifically planned as a function of the property of interest to be extracted, at least after 8–10 years of age (Cirillo et al., 1967; Hatwell, 2003).

Also vision can be considered an exploratory procedure, since it offers an alternative way to explore the object, instead of or in conjunction with haptics (Klatzky et al., 1993). Vision and haptics seem to have similar uni-sensory object processing, potentially supported by common neural substrates. Indeed, cortical areas traditionally considered as specialized for visual processing have been proven to be functionally activated during the corresponding haptic tasks (Lacey et al., 2007). In particular, the lateral occipital cortex (LOC), an object-selective region in the visual pathway, responds consistently also to haptic stimuli (Amedi et al., 2002; Stilla and Sathian, 2008) qualifying it as an area processing geometric shape information independently of the sensory modality used to acquire it (Amedi et al., 2002). These neuroimaging findings are also confirmed by case studies and virtual lesions studies, which indicate the LOC as necessary for both haptic and visual shape perception (Lacey and Sathian, 2014). Additionally, several parietal regions have demonstrated visuo-haptic (VH) responses, as the anterior intraparietal sulcus (aIPS) and the postcentral sulcus (PSC; Stilla and Sathian, 2008). These commonalities would lead to a shared multisensory representation enabling cross-modal object recognition (Lacey et al., 2007).

Though having some similarities, the two modalities are also complementary, as they are not equally efficient in the perception of the different properties of an object. The haptic modality is more appropriate for material properties as hardness, weight or texture, whereas for geometric, spatial properties the visual information seems to be richer and more economical (Hatwell et al., 2003; Gori et al., 2010). Moreover, haptics seem determinant in the development of size perception, whereas vision is crucial in orientation estimation (Gori et al., 2008).

Some differences exist between the two modalities also in the context of memory. Even though perceptual representations can be formed that are sufficiently abstract to permit sharing or exchange across vision and haptics (Easton et al., 1997), haptic working memory is characterized by a more limited and more variable capacity than visual working memory (Bliss and Hämäläinen, 2005). However, long term memory is preserved similarly for objects studied visually and haptically. In particular, when participants are tasked with a recognition test both immediately and after 1 week, the recognition is best for visual study and test, but also haptic memory is still apparent after a week’s delay (Pensky et al., 2008; Hutmacher and Kuhbandner, 2018).

Although it is widely recognized that the strategy of manual and visuo-manual exploration varies as a function of the object property of interest (Lederman and Klatzky, 1987; Hatwell et al., 2003; Kappers and Douw, 2011), it is less clear whether exploration is also prospectively tailored to the use that will be made of the information acquired. Indeed, shape perception is characterized by two different dimensions: *shape encoding*, which includes shape features extraction, online construction and storage of mental representation and *shape matching*, which foresees evaluation of shape features in reference to a stored representation and decision-making (Miquée et al., 2008). The question is whether exploration adaptively changes as a function of its main goal, be it *encoding* or *matching*.

From the few studies which have dissociated these two cognitive processes (Stoeckel et al., 2003; Miquée et al., 2008), it emerges that each stage of haptic shape perception activates a unique set of brain areas. Only a subset of them, those lining the IPS, are recruited throughout the task for encoding, maintaining in memory, and deciding on the shape of tactile objects (Rojas-Hortelano et al., 2014).

Rojas-Hortelano et al. (2014) in particular, used fMRI to measure cortical activation of participants performing a haptic shape discrimination task in which they had to decide whether two objects presented sequentially had the same shape or not. The first exploration of object shape bilaterally engaged the somatosensory, motor, premotor and parietal areas and the primary visual cortex. During the delay phase separating the presentations of the two objects, when participants had to maintain the shape of the first object in short-term memory, the prefrontal cortex (PFC) was active, together with the premotor and the lateral parietal cortices. A control experiment demonstrated further that only the areas in the posterior parietal cortices were specifically engaged in maintaining the short term memory of object shape (and not for instance its temperature). The presentation of the second object, in addition to the mechanisms of exploration and shape encoding that are active also for the first one, engages decision-making processes such as objects comparison and the generation of a decision about whether they are different or the same. These processes recruit a network of frontoparietal areas that include the medial premotor, the right ventrolateral PFC, and the parietal cortices bilaterally, with only the left premotor and the bilateral parietal cortices being specifically engaged for shape-related decision-making processes.

Given the very different neural processing supporting the different phases of shape perception, it might be expected that also the corresponding exploratory behaviors might differ as a function of the sub-goal currently addressed, be it encoding or matching. Indeed, the imaging results by Rojas-Hortelano et al. (2014) suggest that the decision-making process starts as soon as the second object is presented. This could imply that the hand movements used to explore the second object could be aimed not at obtaining its general shape but to obtain information to directly evaluate whether the objects are different or the same, i.e., to support the decision.

This hypothesis could not be thoroughly tested in the above-mentioned studies due to the strong experimental constraints associated with the neuroimaging investigation. Miquée et al. (2008) describe a significant decrease in exploration time for haptic encoding and recall (called “reference” and “comparison” shapes, respectively) of 2D shapes explored with a single finger. The traces of the finger scanning paths show that the global movement patterns are similar in the two phases, but the number of scanning cycles (i.e., finger passages) was reduced in the matching phase. Moreover, the similarity in response times during trials where the two objects were different or equal suggests that the participants made their decision after a thorough exploration of the second object and not as soon as they detected a salient difference between the shape being explored and the memorized reference shape. Rojas-Hortelano et al. (2014) who investigated haptic perception of 3D shapes did not analyze participants’ object motion and fixed also the exploratory time for both encoding and matching phases.

It is, therefore, still to be assessed whether our exploratory behaviors change when we explore an object to encode it in our memory or to recall a memorized information and perform a comparison.

As mentioned above, addressing this question is complex, as currently the characterization of the movements adopted during haptic exploration is performed mainly through lengthy manual annotations by human observers. This approach, beyond being fatiguing for the observers, might also be at risk of missing important exploratory behaviors due to drop in attention or to limitations in visibility (Jansen et al., 2015). It is, therefore, necessary to increase efficiency and reproducibility through new methods for automatic classifications (Jansen et al., 2013, 2015). To this aim, we adopted a novel tool, iCube, a cube which measures its orientation in space and the location of the contacts on its faces and communicates this information wirelessly to a computer. The cube is of about 5 cm side, with 16 tactile cells per face and weighs about 150 g. The main novelty with respect to previous tools is the possibility to investigate haptic exploration with a sensorized object that can be also freely moved in space. The small size and weight allow for a natural manipulation and the sensorization avoids the need for *post hoc* annotations. Although a direct mapping between the data from the cube and the exploratory procedures defined by Lederman and Klatzky, 1987 is not straightforward, as it is not possible to infer exactly the relative motion of the different parts of the body (e.g., of different fingers) with respect to the object, this tool can help augmenting the analysis of exploratory procedure with information about rotation, number and the temporal dynamics of the exerted touches.

With this new tool, here we aim at assessing whether haptic and VH exploratory procedures change during *shape encoding* and *shape matching* tasks. To do so, we positioned little raised pins on the surface of the cube in varying number, in a dice-like configuration, and asked subjects to explore twice the cube, either with vision or while blindfolded, to determine whether the pins configuration varied or not.

Traditionally in same/different haptic tasks, the objects used have different shapes, and as a consequence, the first exploration tends to be slower, as it is necessary to find the contours on which the hands will move. To avoid this potential confound, in this experiment the shape to be explored was kept exactly the same between explorations (a cube) and was well known to participants even before the experiment, with only the pattern of pins on the faces varying. Hence, subjects could decide *a priori* a fixed “cube-exploration” strategy and replicate it during all manipulations, to facilitate their guess.

Conversely, our results show that the exploratory procedures differ significantly when exploration is performed to memorize the cube structure with respect to when it is instead aimed at recalling a previous memorized structure to perform a comparison.

## Materials and Methods

### Subjects

Seventeen subjects took part in the experiments described here [seven males, 10 females, age: 23 ± 2 (SD) years]. All participants but one were right-handed and all were naïve to the goal of the experiment. The research protocol was approved by the Regional Ethical Committee (Comitato Etico Regione Liguria—Sezione 1), all participants provided their written informed consent, received a compensation of 10 euro and followed the same experimental procedure.

### The Device

**Figure 1:**
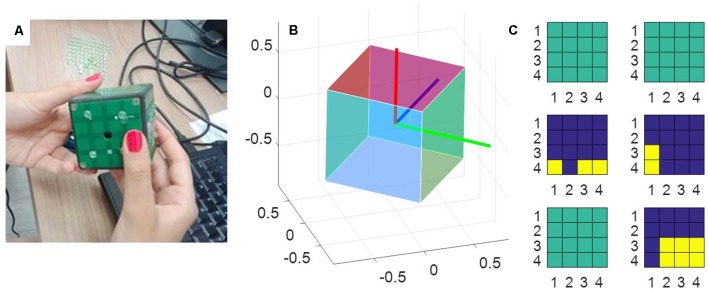
The measurement tool. **(A)** A picture of the iCube with the raised pins positioned on its faces. **(B)** A representation of the cube orientation in space, plotted with MATLAB. **(C)** A snapshot of the activation of the tactile sensors on each of the six faces. Yellow indicates the cells currently touched, blue the cells currently not touched on the same face. Sea green indicates faces that in that instant are totally inactive (i.e., with no cell touched).

The measurement tool used in this study was a sensorized cube designed at IIT, called iCube, which measured its orientation in space and the location of the contacts on its faces and communicated this information wirelessly to a computer. The cube was of about 5 cm side, with 16 cells per face and a weight of about 150 g (see Figure 1). Touch sensing was based on a set of Capacitive Button Controllers (CY8CMBR2016) developed by Cypress Semiconductor Corporation. These were based on Multi Touch technology, enabling detection of simultaneous touches and supported up to 16 capacitive cells (6 × 6 × 0.6 mm), which could be organized in any geometrical format, e.g., in matrix form. Each face of iCube was made with one of these boards. Their sensitivity, i.e., the smallest increase in capacitance that could be detected clearly as a signal, was set to 0.3 pF, so as to allow the device to sense contacts without the need to apply pressure. Orientation estimation was based on a Motion Processing Unit^TM^ (MPU), a nine axes integrated device, combining a three axes MEMS gyro, a three axes MEMS accelerometer, a three axes MEMS magnetometer and Digital Motion Processor^TM^ (DMP). The MPU combined information about acceleration, rotation and the gravitational field in a single flow of data. The information about touches and rotation from iCube were sent to a laptop computer through a serial protocol. The reception was performed through a radio module XBEE together with an integrated circuit developed by FTDI (Future Technology Devices International Ltd., Glasgow, UK) and occurred on average every 348 ms (±52 ms, SD). The communication was constituted of an exchange of properly formatted commands: starting byte, a byte with the address of the board to which the command had been sent, a byte defining the command, one or more optional bytes including the command parameters, the end byte. Through these messages, it was possible to assess the status of the tactile boards and the rotation of the device. These data were further analyzed in MATLAB to extract the pattern of touches, the amount of cube rotation and the speed of rotation (see “Data Analysis” section).

### Protocol

**Figure 2:**
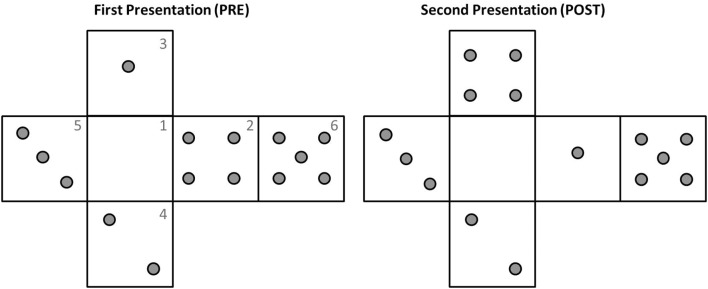
Example of pins configuration during the two explorations of one trial. Note how the pins distributions in faces 3 and 2 have changed from *pre* to *post*. The numbers reported on the faces are indicated here just for illustrative purposes.

Before experiment initiation the device iCube was prepared, connected in wireless mode to a laptop with MATLAB installed and the experimenter positioned on its faces a set of raised plastic gray pins (diameter: 0.5 cm, height: 0.2 cm). The distribution of pins on the cube faces was similar to that of a dice, with each face containing from 0 to 5 pins (see Figure 2 for an example). There was however no limitation of the presence of two equal faces. The participant was comfortably seated in front of the table, where the cube was positioned on a support. Before the experiment subjects were invited to touch and explore the cube to allow for familiarization with the device. In particular, they were asked to try and count the number of pins on the surface of the cube, once with their eyes open and once with eyes closed. The familiarization lasted about 2 min.

In the experimental session participants were asked to explore the cube twice, with the task of understanding whether any change occurred in the pins allocation between the first and the second presentation. All trials, therefore, consisted of a first exploration (*pre*) followed by a second exploration (*post*). The two explorations could be performed either only haptically—with participants wearing a blindfold—or while also looking at the cube (see Table 1). Each of the two trial types was presented to the participants three times in randomized order, for a total of 12 explorations (i.e., six trials) for each subject. The experiment lasted about 25 min on average. At the beginning of each trial subjects received by the experimenter the instructions: e.g., “you will now explore the cube twice, both times with vision (or both times only haptically). Between the two explorations, the pins configuration on the cube might change or remain the same. Please explore the cube as long as you want and then lay the cube on the table and reply either ‘same’ or ‘different.’ ” Then the experimenter handed the cube to the participant. The design was therefore similar to a “study-test” paradigm to assess memory and recall (e.g., Pensky et al., 2008), with the crucial feature of posing no time limit for any phase of the exploration. In the trials with vision, subjects were instructed to keep their eyes closed until they felt the cube touching their hands. In the haptic trial, they wore a blindfold during the whole time.

**Table 1:** Trials organization.

First presentation (*pre*)	Second presentation (*post*)	*CODE*
Haptic	Haptic	*H_1__H_2_*
Visuo-Haptic	Visuo-Haptic	*VH_1__VH_2_*

Between the two phases (*pre* and *post*) the cube could remain exactly the same, but rotated on the support, or could be changed (e.g., by removing or adding one pin to one of the faces or exchanging the pins of two different faces, see Figure 2 for an example). These changes in orientation or pins configuration were rapidly operated by the experimenter, with an interval between explorations lasting on average less than a minute. In 2/3 of the trials, the correct answer was “Different,” in 1/3 of the trials it was “Same.” This uneven distribution was selected to minimize subjects’ fatigue because pilot experimentation indicated that “same” trials were perceived as more difficult.

### Data Analysis

The data about touches and rotations recorded by the iCube were analyzed in MATLAB as described in the following subsections.

#### Touches

From each of the six boards, representing the faces of the cube, the device reported a tactile map, i.e., a matrix of 4 × 4 elements of zeros and one, where one represents a touch. In the analysis we first considered the total number of touches occurred on all the six faces as a measure of tactile exploration. We also computed the exploration duration as the moment between the first and the last touch of the subject (manually cutting for each file the initial phase of recording, when the experimenter put the cube in the hands of the participant).

#### Rotation

The information about the orientation of the cube with respect to its starting position was provided in the form of a quaternion, which was then converted in MATLAB into a rotation matrix to compute instantaneous rotation. The instantaneous angular variation was computed by measuring the angle traversed over time by each of the three unitary axes orthogonal to the faces of the cube (see Figure 1B). In particular, given one *axis*:

$$\text{Δ}{\text{angle}}_{axis}(\text{t})=\text{arctan}\left(\left|\frac{axis(t)\times axis(t-1)}{axis(t)\cdot axis(t-1)}\right|\right)*180{}^{\circ}/\pi$$

We integrated over time the rotations performed by the three axes, to get an estimation of the rotation impressed to the cube in all the possible different directions. To quantify the amount of rotation we considered the maximum value among cumulative sums of the rotations executed by the three axes. The instantaneous rotation speed was instead computed by dividing Δangle _axis_(t) for the corresponding time interval and averaging the results across the three axes and across all the instants in a trial in which the cube was in motion (i.e., angular velocity >1°/s). This selection was done to assess actual velocity of rotation when the rotations were executed, without spuriously reducing the estimate with the analysis of the static phases.

### Statistics

Statistical analysis has been performed on exploration duration, amount of rotation, rotation velocity and the number of touches, averaged among all trials of each condition for every subject. We checked for the presence of outliers, by evaluating whether any subject exceeded the average ±2.5 standard deviations. This happened for two subjects, one in condition H_2_ and the other in almost all conditions. We, therefore, eliminated these participants from the sample for all the subsequent analyses. To assess the difference in exploration due to its goal [memorize the structure (*pre*) vs. recall and compare (*post*)] we ran a repeated measures ANOVA, with TYPE (levels: *pre, post*) and MODALITY (levels: haptic, VH) as factors. A difference has been considered significant for *p* < 0.05.

## Results

To assess whether the exploration is planned differently when aimed at *memorizing* an object—study phase (*pre*)—than when it is used to *recall and compare* with a previously explored stimulus—matching phase (*post*)—we compared different properties of these two different exploratory phases, including features of the tactile exploration and characteristics of the movements actively applied to the cube, both in presence and absence of vision.

### Tactile Exploration

**Figure 3:**
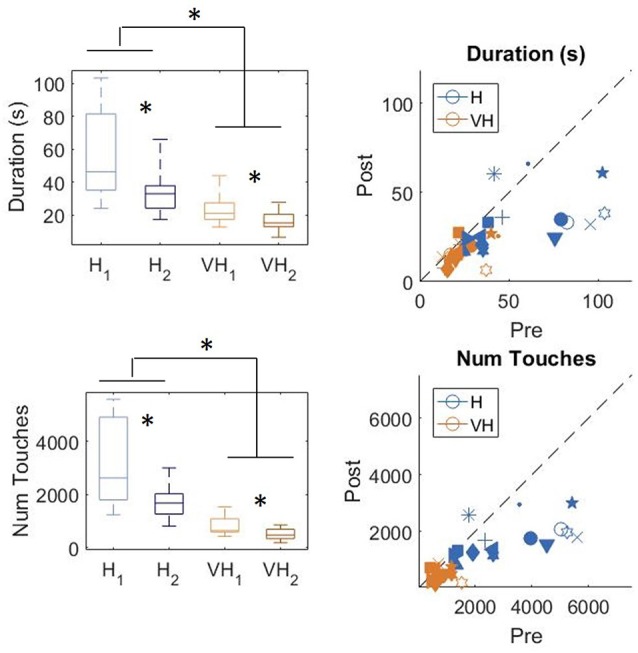
Duration and number of touches. Left panels: distributions of duration (top) and number of touches (bottom) in the Haptic (H) and visuo-haptic (VH) modalities. The subscripts 1 and 2 indicate the first and second exploration of each trial, respectively. On each box, the central mark indicates the median, and the bottom and top edges of the box indicate the 25th and 75th percentiles, respectively. The whiskers extend to the most extreme data points. Asterisks indicate significant difference. Right panel: plot of the individual subjects’ average duration (top) and number of touches (bottom) in the second exploration (*post*) against the same variable in the corresponding first exploration (*pre*), color coded for modality. Different symbols represent different subjects. The dashed black line indicates identity. Elements below the line indicate subjects for which the values in the *post* were lower than in the *pre*.

In Figure 3, top left panel, we show the exploration duration of the first and second explorations for the haptic (H) trials and the VH ones. On average, explorations take longer when performed in the haptic only modality (46.8 ± 5 s SE, Standard Error), than with the help of vision (20 ± 2 s SE) and the difference is significant (two-way repeated measures ANOVA, *F*_(1,14)_ = 41.14, *p* < 0.001). Moreover, in both modalities the first exploration, aimed at memorizing the cube configuration, lasts significantly longer than the second one, for recall and comparison (two-way repeated measures ANOVA, *F*_(1,14)_ = 16.45, *p* = 0.0012). This difference is more accentuated for the Haptic than for the VH trials, as confirmed by a significant interaction (*F*_(1,14)_ = 6.3, *p* = 0.025)^1^. This pattern of results is shown also by the individual data plotted in Figure 3, top right panel. Indeed, the majority of the symbols lie below the black dashed light, indicating a longer exploration in the memorizing phase than in the comparison one. The exact same pattern is replicated when assessing the total number of touches on the cube faces (Figure 3, bottom panels). Haptic exploration entails on average a larger number of touches than VH exploration (two-way repeated measures ANOVA, *F*_(1,14)_ = 63.2, *p* < 0.001) and this number decreases significantly between the first and the second phase of the trials (two-way repeated measures ANOVA, *F*_(1,14)_ = 19.4, *p* < 0.001). Again, the difference between the first and the second exploration is larger for the Haptic than for the VH trials (significant interaction, *F*_(1,14)_ = 12.3, *p* = 0.003).

**Figure 4:**
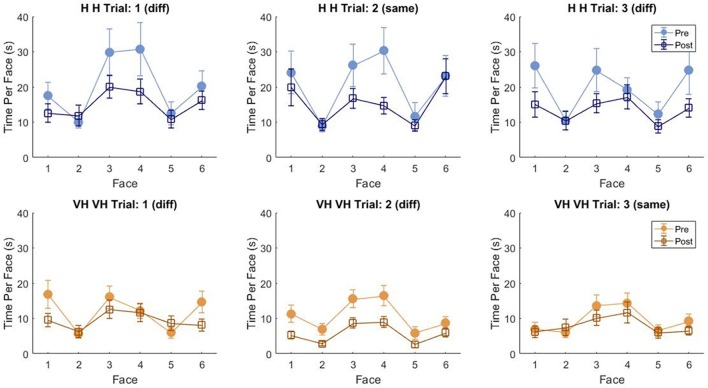
Average touch duration per face. Average time in which each cube face has been touched at least on one cell in one frame during a trial. The three top panels refer to haptic explorations, the three bottom panels to VH exploration. Each panel represents a single trial, with different symbols being associated with *pre* and *post* phases. Error bars indicate standard errors of the mean.

**Figure 5:**
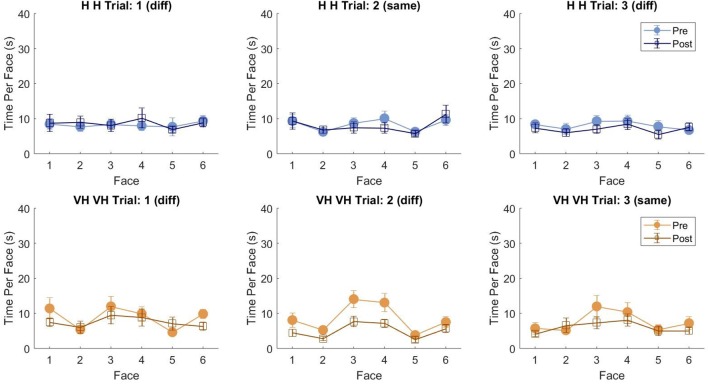
Average touch duration per face (only short touches). Average time in which each cube face has been touched at least on one cell in one frame during a trial excluding all touches which lasted on the same cell for a consecutive period of more than 2 s (steady touches). Same graphical conventions as in Figure 4.

**Figure 6:**
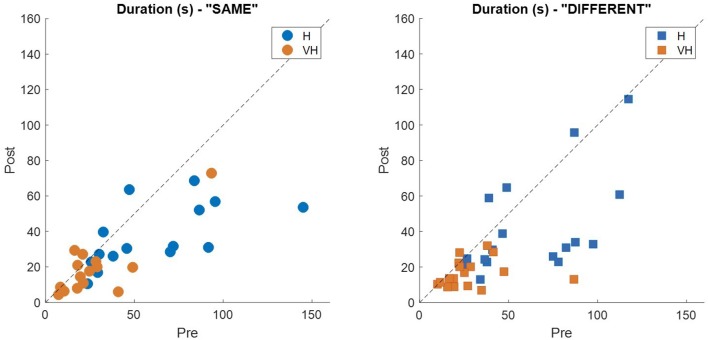
Individual exploration duration. Each panel represents the average exploration duration during the *post* phase plotted against the corresponding duration in the *pre* phase for the “same” trials (left panel) and the “different” ones (right panel). Dashed lines indicate identity.

To assess more in detail how haptic exploration changed as a function of the available sensory modality and of the task phase (memorization and recall) we estimated how long each of the six faces was touched on average in each of the haptic and VH trial, during the *pre* and the *post* phases (Figure 4). At first look, it emerges clearly that individual face exploration is shorter in the VH condition (bottom panels) than in the haptic one (top panels) and that in both modalities the main difference between *pre* and *post* is a general decrease of time. This pattern is similar for both “different” and “same” trials and also among the different faces. There is a tendency in most trials for faces 3 and 4 to be touched for a longer time. Since we are considering all the touches occurred during a trial, here are included also the contacts necessary for holding and rotating the cube and not only the ones purely aimed at exploring the surfaces. If we exclude from this computation all touches which lasted on the same cell for a consecutive period of more than 2 s (steady touches, most probably needed for support), the small temporal differences among faces disappear—in particular for the haptic trials (Figure 5). This suggests that on average participants tended to hold the cube from the back and frontal faces (face id: 3 and 4, respectively) for large parts of the trials while inspecting the other faces either visually or with the other hand as a function of the sensory condition. In summary, in terms of touches distribution, the exploration seems to follow a similar pattern during the encoding and the recall phases of the task, but with a much faster pace in the latter. From a qualitative comparison between behaviors in the “same” and “different” trials, no clear dissimilarities are visible. If participants had on average immediately stopped after finding the different face configuration in the “different” trials, a diverse pattern of time per face distribution would have been expected between these and the “same” ones, where a complete exploration was always necessary. The pattern instead looks remarkably similar, suggesting that in both types of trials, participants tended to analyze all faces in the *post*, before expressing their response. This observation is further confirmed by looking at the total time spent exploring in the *pre* and *post* phases separately for each subject and for “same” and “different” trials (see Figure 6): in both typologies of trials there is a very similar decrease in exploration duration between the encoding and the matching phase.

### Rotation

**Figure 7:**
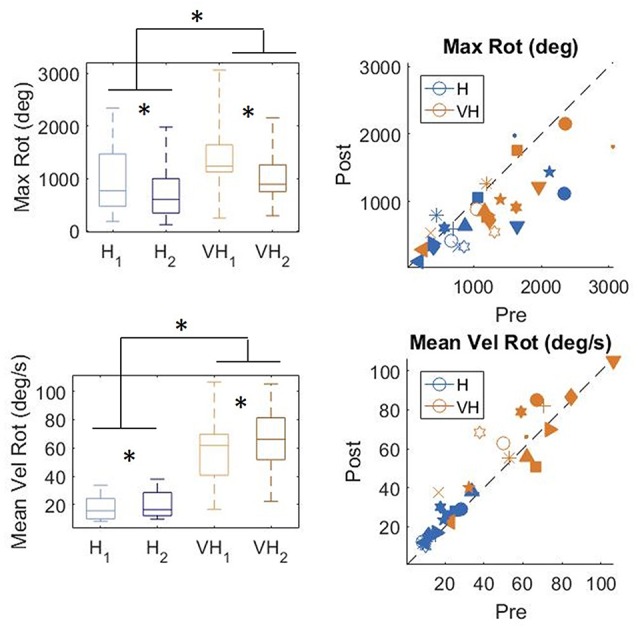
Distribution of amount of cube rotation and average rotation velocity. Left panels: distributions; Right panels: individual data. *indicate significant difference. Same graphical conventions as in Figure 3.

To gain a better insight in the modulation of the exploratory strategy as a function of the goal of the exploration, we analyzed how the cube was moved in the different conditions. In Figure 7, top panels, the distributions of the total maximum amount of rotation is plotted on the left, while on the right individual subjects’ values are presented. In the bottom panels, the corresponding graphs for velocity of rotation are shown. Participants rotated the cube significantly more in the VH condition than when exploring only haptically (two-way repeated measures ANOVA, *F*_(1,14)_ = 17.3, *p* < 0.001). The amount of rotation decreased significantly between the first phase of the trial and the second one (two-way repeated measures ANOVA, *F*_(1,14)_ = 15.98, *p* = 0.0012) and the decrease was similar between the two modalities (two-way repeated measures ANOVA, non-significant interaction *F*_(1,14)_ = 0.36, *p* = 0.56)^2^. The same pattern is visible in the individual data (top right panel).

Rotation velocity (bottom panels) was much faster in the VH condition than when exploration was performed only haptically (two-way repeated measures ANOVA, *F*_(1,14)_ = 62.96, *p* < 0.001). Moreover, it increased significantly during the second exploration (two-way repeated measures ANOVA, *F*_(1,14)_ = 9.4 *p* = 0.008), similarly in both modalities (non-significant interaction *F*_(1,14)_ = 1.18, *p* = 0.29).

Additionally, we computed for each temporal frame which cube face (or faces) were within a specific “cone of visibility.” To do so we estimated the orientation of an hypothetical axis passing through the center of each face, orthogonal to it and outbound oriented, and we computed the angles formed by its’ projections on the frontal and horizontal planes of the absolute frame of reference with respect to the ideal axis connecting the center of the cube starting position and the participant (at the same elevation with respect to the floor). If such angles were inferior or equal to ±45° and the direction of the axis was toward the participant, the face(s) were considered in the “cone of visibility.” This choice was made by observing which faces were in direct view when participants held the cube in their hands. From this analysis, we could determine in which order the faces entered in the “cone” in different trials and whether there were sequences of orientations (or transitions) more frequent than others.

To do that we extracted a transition matrix: a 6 by 6 matrix in which each element corresponds to the number of times in which the transition has occurred between the face individuated by the row number and the face corresponding to the column number. For instance, the cell in row 2 and column 3 reports the number of times in which at first face 2 was in the cone of visibility and then face 3 entered the cone. An element in the diagonal instead would indicate for how many frames the same face was maintained consecutively in the cone of visibility.

For each subject we computed one matrix for *pre* and *post* for both the Haptic and the VH conditions, by summing the matrixes of all trials for each specific condition. We then normalized all cells dividing them by the maximum value of each matrix, leading to values ranging between 0 and 1, making each cell a proportion of the maximum amount of transitions occurred.

**Figure 8:**
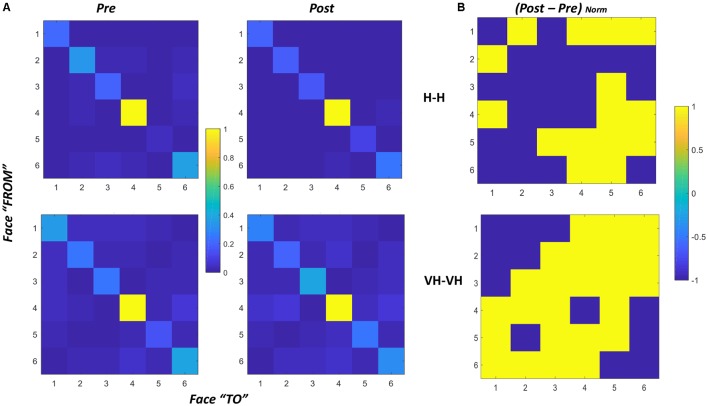
Transition matrices. **(A)** Transition matrices for *pre* and *post* trials, for Haptic (top) and VH (bottom) conditions for all subjects. Each cell represents the proportion of transitions from one face (row id) to the other (column id) in the cone of visibility. The proportion is computed with respect to the maximum of the matrix. Cells on the diagonal reflect the proportion of time instants in which the same face was maintained in the cone. **(B)** Normalized difference between the *post* and *pre* matrixes for Haptic (top) and VH (bottom) conditions. Values have been set to 1 when positive, −1 when null or negative. As a result, yellow cells indicate transitions which proportionally increased from *pre* to *post*.

We also derived a single matrix for each condition, by summing over all the subjects the matrixes of all trials for each specific condition and normalizing the result. The derived matrixes are reported in Figure 8, panel A. To evaluate potential differences in proportion of transitions between *pre* and *post* sessions we additionally computed the difference between the two sessions for both the Haptic and the VH conditions (Figure 8, panel B).

In all graphs of panel A, it is clear that the face which remained most consecutively in the cone of visibility is face 4, which is actually the face which is handed toward the participant by the experimenter in most trials. Then, for all the faces there is a certain amount of time in which they are kept consecutively in a similar orientation toward the participant. The pattern is remarkably similar across all conditions, suggesting that even though the number of transitions might differ between different modalities, there is a tendency to maintain all the faces stable for a similar proportion of time during exploration.

The difference between transitions in the Haptic and in the VH condition is evident outside the diagonal: with vision available a lot more of transitions occur between different faces, and this phenomenon seems to increase further in the *post* session with respect to the *pre*. Conversely, in the Haptic condition, the proportion of transitions occurring between different faces is much smaller than the maintenance of a single face in the same orientation and seems to remain almost unchanged between the *pre* and the *post* conditions. The larger increase of transitions between different faces in the *post* VH condition outside the diagonal is significant ($X_{\left(1,N\;=\;60\right)}^{2}$ = 4.44, *p* = 0.035) and can be visualized in the larger number of yellow off-diagonal cells in Figure 8, panel B, top graph, if compared with the corresponding Haptic matrix (bottom graph).

To verify this observation, we computed for each subject the difference in proportion of transitions between the *pre* and the *post* conditions, by subtracting the corresponding matrixes. We then counted the number of cells with positive values in the resulting difference matrix, considering only the 30 cells outside the diagonal. Positive values correspond to the transitions for which the proportion of occurrence increased from the *pre* to the *post* trials. In the VH conditions about 36% (±14%) of such transitions increase, whereas in the Haptic conditions only about 18% (±14%) of the transitions increase and this difference is significant (paired sample *t*-test *t*_(14)_ = −3.6, *p* = 0.003).

This might be interpreted suggesting that a difference in exploratory patterns occurs between *pre* and *post* sessions and that such change differs between Haptic and VH explorations. With vision, participants perform more rotations of the cube from one face the other corresponding to the large number of transitions (outside the diagonal). Moreover, this phenomenon tends to increase in the *post* session, where probably participants attempt to check the evaluations and maps they built in the *pre* session, by assessing relative positions of different configurations of pins, rapidly rotating the cube in all possible configurations and also exploiting orientations that give the possibility to glance at more faces at the same time (i.e., looking from a vertex, enabling the vision of two or even three faces at the same time). Conversely in the Haptic condition—especially in the *post* session—it seems that participants tend to select a reduced number of fixed rotations (e.g., switching from face 4 in front to face 6 and then vice versa) and to keep the resulting position, to be able to then explore the cube faces by moving the hands over them with a relatively stable spatial frame of reference.

## Discussion

This work shows for the first time that even in presence of a well-known object, humans change the way they explore it, when they manipulate it to encode what is on its faces, vs. when they manipulate it again to recall it and make a comparison with the first exploration. Not only the latter process becomes faster and involves fewer touches, but it also entails faster and different rotations. Hence, memorization and recall are not only processed by different brain areas (Rojas-Hortelano et al., 2014) but are supported by significantly different behavioral patterns, suggesting a tuning of the exploratory plans guided by the current action goal.

Interestingly, this marked difference in exploration between the two tasks is clearly present for both Haptic and VH exploration, though being more accentuated for the haptic-only condition. Hence, even when vision is available to guide manipulation, still the two explorations differ significantly. This suggests that memorization requires more effort independently of the modality with which exploration is performed and not only when it is limited to just manual analysis.

Crucially, our experimental design allowed unlimited time for both exploration and recall, leaving to participants the free decision of how (and how long) to explore the object in both phases. As a result, they could take advantage of the free time to thoroughly explore the cube in both presentations to facilitate the recognition. Indeed, replicating one-to-one the same exploratory strategy twice (both in *pre* and *post*) could *a priori* represent a reasonable method to simplify the recognition of similarities and differences among the two presentations. Conversely, most participants tailored their exploration to the different nature of the task (memorizing vs. recall) performing the two explorations differently, not only in terms of timing, but also in the number of touches and the amount, type and speed of rotations. Interestingly, the reduction in exploration time was observed also in the “Same” trials, in which the cube was not modified between the two explorations. So, even when visiting the whole cube was necessary to provide an accurate response, the *post* exploration was performed differently from the *pre* one. In summary, uni- and bimodal exploratory strategies are modified as a function of the goal of the exploration, i.e., memorization or recall, even when the properties to be analyzed in the object and the sensory modalities available are kept constant.

Additionally, the results demonstrated that the availability of vision—together with haptics—substantially changes how the object is manipulated, leading to faster decisions, faster and larger rotations and a reduced number of touches. The better temporal performance observed in the VH condition hints to a higher efficiency of simultaneous visual and haptic exploration with respect to unimodal haptic exploration. This finding extends previous results obtained in classical geometric shapes recognition tasks (Hatwell et al., 2003). The reduction in the number of touches is consistent with the role of vision as a guide, providing a quick “preview” of the object properties, and limiting the instantiation of an extensive haptic exploration when the visual encoding is sufficient to give a response (Klatzky et al., 1993). It is worth noting that notwithstanding the reduction in the total number of touches in the VH condition, in our experiment participants still performed several touches, which might suggest that touch was used not only to support cube motion, but also to actively gather haptic information, in support to visual inspection. A relevant contribution of haptic exploration to the response, even in the presence of vision, might be due to the property of the stimulus to be assessed. Indeed, the analysis of the pins configuration can be interpreted as a form of texture discrimination rather than a pure geometrical task (as shape recognition) and haptic sensing is particularly efficient for texture perception (Jones and O’Neil, 1985).

The addition of vision had an opposite effect on how the object was touched and how it was rotated. Indeed, while the number of touches decreased, the amount of cube rotations and their speed significantly increased from the Haptic to the VH condition. This suggests a guidance of vision in the selection of the responses. While haptically it is possible to explore simultaneously different faces of the cube, as the front and the back, with fingers and thumbs respectively (Newell et al., 2001), with no need for a complete rotation of the object, vision requires that all faces are positioned so as to allow for a visual inspection, inducing subjects to perform larger rotations. These rotations can, however, be performed at a very fast pace, as they are mainly finalized to put each face in better view rather than being part of the strategy for tactual exploration. As a result, the VH recognition is significantly faster than the Haptic one, even if it involves larger rotations of the object to be analyzed.

The current experimental setting has some limitations, in that it provides only an object-centered description of the touches and rotations occurred, without allowing to understand exactly how visual analysis and haptic exploration are coordinated. Another factor that the current investigation did not address is how the bimanual coordination unfolds during the exploration. The discussion of the relative roles of the right and the left hand in dichaptic exploration is still controversial, with some evidence in favor of a higher sensitivity of the left hand—e.g., for curvature (Squeri et al., 2012) or for geometrical shape discrimination (Fagot et al., 1997), but depending on a variety of factors, as gender, type of shape, exploratory approach (Streri, 2003). The analysis described here clearly shows how also the presence of vision and the memorization goal are determining factors in the planning of the bimanual exploratory strategy adopted by participants.

Notwithstanding these limitations, the current study has demonstrated that exploratory strategies are not only tailored to the property to be extracted—as described in the traditional Exploratory Procedures classification—but are finely tuned to the current goal of the shape perception task (memorize/encode vs. or recall/match).

Shedding light on how different factors shape haptic exploration and how this is connected with efficient perception of object properties could help develop novel training protocols designed to help participants showing perceptual or memory deficits or to support learning during development. Multiple evidence suggests that haptic and VH exploration can facilitate letters and shapes understanding in young children (Bara et al., 2004; Kalenine et al., 2011). As a consequence, teachers and occupational therapy practitioners often engage children in multi-sensory experiences as part of teaching or treatment, respectively (Coté, 2013). A better understanding of how haptic exploration is tailored by sensory-motor and task constraints could help in appropriately designing these activities to maximize their impact. Moreover, a systematic modeling of the features of efficient exploration in healthy individual could also allow detecting the occurrence of abnormal exploratory behaviors that emerge during life—either due to developmental changes or to the set in of a disease. For instance, Mild Cognitive Impairment leads to significant deficits in haptic tasks (Grunwald et al., 2002), among the spectrum of deterioration of memory and perceptual-motor capabilities associated with this condition. In contrast, haptic memory has been shown to be very well preserved in healthy ageing (Sebastián et al., 2011), while being even more compromised in patients with dementia—e.g., Alzheimer disease (Ballesteros and Reales, 2004). A simple and non-invasive procedure providing information on the manipulation strategy adopted during haptic memory tasks could represent a valid addition to the assessment measures currently in place, supporting the quantitative evaluation of both perceptuo-motor skills and memory processes.

We also posit that the use of simple noninvasive tools as the sensorized object described here could be in future used to augment the automatic assessment of haptic exploration strategies, reducing the need of manual annotation of videos, to increase reproducibility of the measures, a currently crucial challenge in the context of haptic analysis (Jansen et al., 2013, 2015). Indeed, the automatic extraction of the temporal dynamics of the exerted touches could allow reconstructing on average the effector’s motion features, together with the cube rotation. This information could in future be used to derive similarities and differences with those exploratory procedures which are principally defined by the kinematics of the exploring hand.

## Ethics Statement

The research protocol was approved by the Regional Ethical Committee (Comitato Etico Regione Liguria—Sezione 1), all participants provided their written informed consent, received a compensation of 10 euro and followed the same experimental procedure.

## Author Contributions

All authors contributed to the design of the experiment. MA and FD cured the data collection. AS, MA and FD cured the data analysis. AS and GS contributed to the writing of the manuscript. All authors revised the manuscript.

## Conflict of Interest Statement

The authors declare that the research was conducted in the absence of any commercial or financial relationships that could be construed as a potential conflict of interest.

## Footnotes

^1^Since a Lilliefors test indicated that the Durations measured in the first visuo-haptic exploration (VH_1_) were distributed non-normally (*p* = 0.019), we tested all differences related to Visuo-Haptic duration also with Wilcoxon signed rank tests, which confirmed the reported results. In particular, the first exploration lasts longer than the second one (paired *t*-test, *p* = 0.003 for Haptic, Wilcoxon signed rank *p* = 0.001 for Visuo-Haptic) and this difference is more accentuated for the Haptic than for the Visuo-Haptic trials (Wilcoxon signed rank test on the differences H_2_−H_1_ vs. VH_2_−VH_1_; *p* = 0.018). All differences are significant after Bonferroni correction.

^2^Since the rotations were not distributed normally in the two Haptic explorations (Lilliefors tests, *p* = 0.034 and *p* = 0.037, respectively), we replicated the analysis with non-parametric tests for all comparison related to Haptic Amount of Rotation. The decrease in amount of rotation in the Haptic modality did not reach significance (after Bonferroni correction: Wilcoxon signed rank for Haptic, *p* = 0.035), but the decrease was similar between the two modalities (Wilcoxon signed rank test on the differences H_2_−H_1_ vs. VH_2_−VH_1_: *p* = 0.6787).
